# Healthcare providers’ perspectives on implementing a brief physical activity and diet intervention within a primary care smoking cessation program: a qualitative study

**DOI:** 10.1186/s12875-023-02259-3

**Published:** 2024-01-06

**Authors:** Nadia Minian, Kamna Mehra, Mathangee Lingam, Rosa Dragonetti, Scott Veldhuizen, Laurie Zawertailo, Wayne K. deRuiter, Osnat C. Melamed, Rahim Moineddin, Kevin E. Thorpe, Valerie H. Taylor, Margaret Hahn, Peter Selby

**Affiliations:** 1 INTREPID Lab, Centre for Addiction and Mental Health, Toronto, Canada; 2 Department of Family and Community Medicine, University of Toronto, Toronto, Canada; 3 Campbell Family Mental Health Research Institute, IMHPR, Centre for Addiction and Mental Health, Toronto, Canada; 4 Department of Pharmacology and Toxicology, University of Toronto, Toronto, Canada; 5 Institute of Medical Sciences, University of Toronto, Toronto, Canada; 6 Applied Health Research Centre, Li Ka Shing Knowledge Institute, St Michael’s Hospital, Toronto, Toronto Canada; 7 Dalla Lana School of Public Health, University of Toronto, Toronto, Toronto Canada; 8 Department of Psychiatry, Hotchkiss Brain Institute, University of Calgary, Calgary, Canada; 9 Department of Psychiatry, University of Toronto, Toronto, Canada; 10 Schizophrenia Division, Centre for Addiction and Mental Health, Toronto, Canada; 11 Banting and Best Diabetes Centre, University of Toronto, Toronto, Canada

**Keywords:** Smoking cessation, Physical activity, Exercise, Diet, Fruit/vegetable consumption, Implementation, Health behaviour

## Abstract

**Background:**

Post-smoking-cessation weight gain can be a major barrier to quitting smoking; however, adding behavior change interventions for physical activity (PA) and diet may adversely affect smoking cessation outcomes. The “Picking up the PACE (Promoting and Accelerating Change through Empowerment)” study assessed change in PA, fruit/vegetable consumption, and smoking cessation by providing a clinical decision support system for healthcare providers to utilize at the intake appointment, and found no significant change in PA, fruits/vegetable consumption, or smoking cessation. The objective of this qualitative study was to explore the factors affecting the implementation of the intervention and contextualize the quantitative results.

**Methods:**

Twenty-five semi-structured interviews were conducted with healthcare providers, using questions based on the National Implementation Research Network’s Hexagon Tool. The data were analyzed using the framework’s standard analysis approach.

**Results:**

Most healthcare providers reported a need to address PA and fruit/vegetable consumption in patients trying to quit smoking, and several acknowledged that the intervention was a good fit since exercise and diet could improve smoking cessation outcomes. However, many healthcare providers mentioned the need to explain the fit to the patients. Social determinants of health (e.g., low income, food insecurity) were brought up as barriers to the implementation of the intervention by a majority of healthcare providers. Most healthcare providers recognized training as a facilitator to the implementation, but time was mentioned as a barrier by many of healthcare providers. Majority of healthcare providers mentioned allied health professionals (e.g., dieticians, physiotherapists) supported the implementation of the PACE intervention. However, most healthcare providers reported a need for individualized approach and adaptation of the intervention based on the patients’ needs when implementing the intervention. The COVID-19 pandemic was found to impact the implementation of the PACE intervention based on the Hexagon Tool indicators.

**Conclusion:**

There appears to be a need to utilize a flexible approach when addressing PA and fruit/vegetable consumption within a smoking cessation program, based on the context of clinic, the patients’ it is serving, and their life circumstances. Healthcare providers need support and external resources to implement this particular intervention.

**Name of the registry:**

Clinicaltrials.gov.

**Trial registration number:**

NCT04223336.

**Date of registration:**

7 January 2020 Retrospectively registered.

**URL of trial registry record:**

https://classic.clinicaltrials.gov/ct2/show/NCT04223336.

**Supplementary Information:**

The online version contains supplementary material available at 10.1186/s12875-023-02259-3.

## Background

Tobacco use, physical inactivity, and low fruit/vegetable consumption are important risk factors for cardiovascular disease, diabetes, cancer, and other conditions [1–10] and contribute to a substantial proportion of preventable death [11]. Roughly 90% of the Canadian population has at least one of these health risk behaviours [12–14]. Research has shown that these health risk behaviours may interact with each other, for example, physical activity (PA) may help in smoking cessation by reducing cravings [7, 15–18], and PA and diet considerations may affect post-cessation weight gain, which is a major barrier to quitting smoking [19–21]. Brief interventions have been shown to increase PA and/or fruit/vegetable consumption [22, 23]. As such, smoking cessation programs may provide an opportunity to address these multiple modifiable health risk behaviours through brief intervention approaches. However, there is a risk that the addition of generic brief interventions focused on weight management might reduce smoking cessation rates by about 40% while being ineffective [24]. The challenge of addressing multiple health behaviours in primary care settings is not well described. We conducted the “Picking up the PACE (Promoting and Accelerating Change through Empowerment)” trial between November 2019 to May 2021, a Hybrid Type 1 trial, within a mixed methods sequential quantitative-qualitative study to evaluate the impact of the addition of a co-created brief intervention to increase PA and fruit/vegetable consumption at the point of care in an ongoing Smoking Treatment for Ontario Patients (STOP) smoking cessation program in primary care settings in Ontario, Canada [25–27]. The STOP program provides behavioural counseling and nicotine replacement therapy at no cost to the participants and the STOP Portal is a web-enabled data collection and treatment management tool used by all healthcare providers in the program to enroll their patients. This portal includes a clinical decision support system that provides actionable information to healthcare providers to support clinical care, such as, a brief intervention for at-risk alcohol use [28] and managing mood for those experiencing depressive symptoms [29]. Healthcare providers were trained in the brief intervention through an interactive webinar [30, 31]. Additional information about the development of the intervention and the training webinar was described previously [25].

The intervention consisted of screening patients for their levels of PA and fruit/vegetable consumption based on Canadian guidelines [32, 33], alerting healthcare providers when patients did not meet criteria for adequate levels of PA (150 min of moderate-to-vigorous-intensity aerobic PA per week) or fruit/vegetable consumption (7–8 servings of fruits/vegetables per day), and guiding healthcare providers about positively communicating the impact of low PA and/or fruit/vegetable consumption on patients’ health and smoking cessation, as well as providing a self-monitoring resource to patients to track their exercise and diet (i.e., a tracking sheet) [25]. A randomized controlled trial (RCT) compared this intervention to a control group of healthcare providers whose patients were screened, but who did not receive the alert, guidance about risk communication, or the self-monitoring resource. This trial showed that the intervention did not significantly change PA, diet, or smoking status [26]. Healthcare provider perspectives about implementation can be essential to understand if the intervention will be adopted and what determinants need to be addressed to ensure effective uptake and provision to clients. Other studies have focused on healthcare provider perspectives about PA and/or diet in a variety of settings [34–36], however, this is the first study, to the best of our knowledge, to explore factors affecting implementation within a primary smoking cessation program.

The COVID-19 pandemic was declared four months after the introduction of the PACE trial, and the healthcare providers had to pivot to virtual (phone or video) communication to provide the smoking cessation intervention [37], and the recommendations for PA and fruit/vegetable consumption [26]. We previously documented how the pandemic affected the STOP program [37, 38], however these studies did not explore how the pandemic affected the implementation of the PACE trial.

The current study was conducted to explore the factors that affected the implementation of the PACE trial among primary care healthcare providers, including how COVID-19 affected its implementation, and understand the context of the trial’s quantitative findings.

## Methods

### Aim and design

In order to understand the factors that affected the implementation of this intervention and contextualize the findings of the RCT, as well as to assess the impact of COVID-19 on the implementation process, we conducted semi-structured interviews with healthcare providers. The interview questions were guided by the National Implementation Research Network’s Hexagon Tool [39, 40]. This tool comprises six indicators in two domains: implementation site indicators (need, fit, and capacity) and program indicators (supports, usability, and evidence). These indicators ensure comprehensive assessment of the fit and feasibility of an intervention [41].

### Participants

A purposive non-probabilistic sampling [42] was conducted where healthcare providers from three different types of organizations (Family Health Team (FHT), Community Health Centre (CHC), and Nurse Practitioner-Led Clinic (NPLC)) were recruited if they had seen at least four patients who had not met the national guidelines for PA and fruit/vegetable consumption. This cut-off of four patients was selected to ensure sampling from healthcare providers who had sufficient experience with the clinical decision support system. Among each type of organization, healthcare providers were organized based on the number of clients who had low levels of PA and fruit/vegetable consumption. The healthcare providers with most number of clients were contacted first, and if they did not respond, those next on the list were contacted. Recruitment for the interview was conducted via telephone by a research assistant who did not have any previous relationship with the healthcare providers, with up to three phone attempts made for each prospective healthcare provider. By the third attempt, if the research assistant was unable to connect with the prospective healthcare provider via phone, they would send the recruitment invitation via email.

Ethics approval was obtained from the Centre for Addiction and Mental Health Research Ethics Board (REB #119/2018) and all healthcare providers provided informed consent before participating in the interview. Data was de-identified and quotes were presented using numerical codes to protect participant’s privacy and confidentiality.

### Data collection

The development of the interview guide was structured around the six indicators of the Hexagon Tool, and each question within the interview guide addressed at least one of the tool’s six indicators (Additional File 1). All interviews were conducted via telephone between January 2021 and May 2021 by a research staff member, with each interview lasting approximately an hour. Interviews were audio-recorded and transcribed verbatim by a transcriptionist and the audio files and the interview files were cross-checked and verified for accuracy by a researcher.

### Data analysis

The framework analysis approach, a qualitative analysis method described by Gale et al. (2013) [43], was utilized to analyze the data following these seven steps: transcription, familiarization with interview, coding, developing a working analytical framework, applying the analytical framework, and charting data into the framework matrix. This approach was adopted since it allows data analysis based on a pre-existing framework such as the Hexagon Tool, and allows for both deductive and inductive analysis. NVivo was utilized to support the framework analysis.

After listening to the audio recordings and reading all the transcripts, five transcripts were chosen for initial coding, and a preliminary codebook was iteratively developed using the Hexagon Tool, the interview guide, and coding these five interviews. We chose transcripts that had complex data and would prompt extensive coding. Two coders (KM and ML) used the preliminary codebook, which had codes about the six Hexagon Tool indicators, impact of the COVID-19 pandemic, impact of randomization on the levels of PA and fruit/vegetable consumption, and impact of the intervention on smoking cessation, to code these 5 transcripts. Following this initial coding, three researchers (KM, ML, and NM) conducted minor modifications to the codebook. Two new codes were added (e.g., Organization culture conducive to PACE intervention) and three codes were changed. Specifically, during the iterative process of developing the codebook, we realized that there was substantial convergence between the indicators of “fit” and “evidence.” When participants talked about the “evidence” for the PACE intervention, they also mentioned that this made it a good “fit”. Thus, we decided to amalgamate these two themes into a singular thematic category denoted as “fit/evidence.” Once these five transcripts were coded (by KM and ML), we checked for inter-coder reliability using the coding comparison query in NVivo found an agreement of 98% between the two coders. This codebook was then used to code the remaining 20 interviews by only one researcher (KM). This followed the recommendation that at least 10% of the interviews are coded by two coders [44].

Once all transcripts were coded, a framework matrix was developed using the NVivo function, with the cases (healthcare providers) and codes organized into rows and columns, respectively. The data within the intersecting cells summarized the content for that practitioner and theme. This matrix was then used to conduct the content analysis portion of framework analysis. The most representative quotes from each theme were included in this paper. A reflexive approach was adopted by the research team (some of whom had a dual role of clinicians and researchers) by acknowledging their training, education and working status in the organization that coordinates the STOP program while analyzing the data. In addition, sub-analyses of the data was conducted based on the training received by the healthcare providers, and the COVID-19 data was sub-analyzed using the Hexagon Tool indicators. In this manuscript, we report the themes that were endorsed by five or more (20%) participants. This manuscript fulfills the criteria from the Relevance, Appropriateness, Transparency, and Soundness (RATS) guidelines for reporting qualitative research [45] (Additional File 2).

## Results

Out of 49 healthcare providers invited to the study, 26 agreed to participate and 25 completed the interview (one healthcare provider was unable to conduct the interview due to scheduling conflicts), resulting in a response rate of 51%. The baseline characteristics of the healthcare providers are presented in Table 1.

**Table 1:** Baseline characteristics of the 25 healthcare providers who participated in the interviews

Baseline characteristics	N (%)
Organization type	
• Community Health Centre	9 (36%)
• Family Health Team	10 (40%)
• Nurse Practitioner Led Clinic	6 (24%)
Healthcare providers occupation	
• Nurse	14 (56%)
• Pharmacist	3 (12%)
• Respiratory Therapist	2 (8%)
• STOP Program Coordinator	2 (8%)
• Health Promoter	2 (8%)
• Community Health Worker	1 (4%)
• Social Worker	1 (4%)
Years practitioner has been involved in STOP program	
• < 2 years	1 (4%)
• 2–5 years	13 (52%)
• > 5 years	11 (44%)

Note: STOP = Smoking Treatment for Ontario Patients

Most of the healthcare providers (n = 15, 60%) had completed general training with respect to PA and fruit/vegetable consumption (e.g., through their nursing training), some had attended the webinar that provided specific training about PA and fruit/vegetable consumption (n = 6, 24%), and some reported no specific training with respect to PA and fruit/vegetable consumption (n = 4, 16%). Notably, this variability did not manifest in healthcare providers reporting distinct barriers or facilitators.

Below, we present our findings organized according to the six key indicators of the Hexagon Tool.

### Need: Healthcare providers’ perception that the PACE intervention is relevant for the patients

The majority of the healthcare providers found the PACE intervention to be relevant for STOP clients (n = 19).

“It’s certainly relevant. It’s an important part of living a healthy life. It’s an important strategy for trying to reduce smoking.” – Interview 5.

“It’s definitely relevant especially the physical activity one because physical activity changes your outlook and can really help, in many ways, to avoid smoking, so that’s definitely relevant. And as far as the diet, somebody who has a healthy diet often will be more motivated to be healthy in other ways, so it’s pretty relevant.” – Interview 14.

Six healthcare providers mentioned that patients had an increased need for an intervention for PA and fruit/vegetable consumption due to the COVID-19 pandemic, for example, due to their inability to go out for exercise, worsened eating habits, or gaining weight.

“I find that exercise has definitely decreased, because we’re all supposed to be inside and we’re not supposed to be going out doing anything.” - Interview 25.

### Fit/evidence: Healthcare providers’ perception of the practice-based evidence of usefulness and fit of the intervention with clients’ and the program’s values

Several healthcare providers mentioned that there was evidence of PA and fruit/vegetable consumption being connected with (n = 16) or helping with smoking cessation (n = 12) and thus being a good fit with the STOP Program (n = 19).

“Well, one of the things, of course, would be a lot of expressed concern about weight gain when trying to quit smoking… also to try and assist them in looking at healthier ways of dealing with stress like through activity… helping them with cravings. Using physical activity as a diversion for cravings. And also, to develop an awareness of eating habits, they may try substituting food, but teaching them that it’s okay, you can substitute, but choose healthy fruits and vegetables… cut up raw vegetables as a substitute or an alternative to the cigarettes.” – Interview 7.

“I still encourage them to try to get fruits and vegetables because I say it will give you more energy to help you quit. I explain that when you’re dragged out because you’re not getting enough food to eat, then you’re going to be less able to quit.” – Interview 13.

However, most healthcare providers, (n = 15), mentioned the need to counsel patients on the benefits of a holistic approach to smoking cessation, as many patients did not see the PACE intervention as aligning with their expectations when quitting smoking.

“I find a lot of them are receptive when you start to explain how these are all related, which seems to make a little bit more sense to them. Sometimes they respond by say like, why are you asking all these questions? And what does that have to do with my smoking? … I think it’s just the idea of reinforcing how all of our health behaviours are really interconnected and how, when we make a change in one area of our life, that it can have that snowball effect in other areas of our life too.” – Interview 10.

“Patients might have a bit of a harder time feeling like it’s relevant until we kind of explain that multifactorial approach and kind of that all around healthcare.” – Interview 18.

The majority of healthcare providers (n = 20) indicated that various social determinants of health influenced the suitability of the intervention. For example, they felt it was inappropriate to provide the intervention to patients with low income or food insecurity as they were important barriers to clients’ ability to adopt the intervention.

“Well, once again, it’s just…it’s financial. They don’t have the opportunity to get a gym pass or things that other clients would be able to get probably… You can prompt all you want, but if they don’t have the ability to buy healthier foods, or know how to cook properly, or be able to go to a gym, it doesn’t matter how much prompts come up, they can’t do it.” – Interview 17.

“Sometimes if I’ll ask ‘what do you think is stopping you from eating more fruits and vegetables?’, they’ll always say it’s the cost… especially in Northern Ontario, the cost of produce is even more expensive because of the fees associated with transporting the produce here… so that does seem to be a barrier for a lot of people.” – Interview 10.

“I have clients that don’t, a) have access to computers, b) that are illiterate, like, don’t have a high degree of education.” – Interview 11.

Similarly, clients’ mental health and/or substance use would also act as barriers to healthcare providers’ implementation of the PACE intervention (n = 6).

“If someone’s struggling with severe addiction of other substances with tobacco or severe mental health issues, sometimes I find it harder to deliver the PACE as effectively because of the fact, not because it’s not important to do, but because of the fact that there’s other things that often also need to be addressed. Like, you know, risk of self-harm and different things like that that I need to make sure I spend the time on.” – Interview 5.

A majority of healthcare providers (n = 20) reported that very few clients prioritized PA and fruit/vegetable consumption, mainly because their focus was on smoking cessation. In addition, 11 healthcare providers mentioned that the intervention was not a good fit with the STOP Program as it was overwhelming for some/many of their clients or for them.

“… even concentrating on the smoking cessation, it’s just about all they can do at the time. They’re really not ready. They’re not prepared for talking about changing their whole diet or physical activity again. I think they’re trying to stay very focused on just the smoking cessation. It’s just too much for them at one time.” – Interview 17.

“A lot of people, they don’t want to look at their physical activity and they don’t want to look at their drinking while they’re trying to quit smoking. They just…if they want to quit smoking, that’s what they want to do. They don’t want to worry about other aspects. Even though we know that it’s all connected, sometimes it’s hard for them to be able to wrap their head around so many changes.” – Interview 24.

“I find too, by the time I do the interview, the paperwork like, everything, I’m actually pretty exhausted too…” – Interview 11.

Only five healthcare providers commented on the role of organizational culture, reporting that it was conducive to implementing the PA and fruit/vegetable consumption intervention.

“I feel like we have a…health promotion has really become kind of just like a culture here. It’s not just something that I’ll do as a health promoter, but it’s something that, I think all of our providers here are encouraged and are passionate to talk their patients about.” – Interview 10.

Eight healthcare providers mentioned that the pandemic impacted patients’ social determinants of health (e.g., lower financial stability) or mental health (e.g., depression, higher stress) and this had negative impacts on how well the PACE intervention fitted into patients’ lives.

“It’s hard right now with COVID. It’s just…some people just aren’t motivated to do much, and it’s really taken a hit on mental health.” – Interview 20.

### Capacity: Healthcare providers’ capacity to implement the intervention based on their role, training, and workflow

A majority of healthcare providers (n = 21) mentioned that their role or training (e.g., health promotion training or nursing training) facilitated the communication with clients about PA and fruit/vegetable consumption.

“I think that because of my background I may have had a bit of an advantage because, again, just focusing on chronic disease prevention and health promotion, again, I’m used to discussing these types of behaviours and the whole goal-setting aspect with patients. So, I think that made it a little bit easier for me when you did introduce this as part of the STOP Program.” – Interview 10.

“I believe that nurses have a little knowledge about a lot of things, and so we touch into things like nutrition, and we touch into things like exercise and depression.” – Interview 24.

However, 15 healthcare providers mentioned that time was a barrier when conducting the intake session and asking questions about PA and fruit/vegetable consumption.

“Um, sometimes time is a factor, right? If I know that time is limited for myself, if I have patients that are booked, and it’s one after the other and I don’t have time to go over in the call, then… those are things where… I just won’t be able to take as much time.” – Interview 5.

“Well, it depends, because sometimes the baseline form can take a long time to fill out and it just depends how much time they have and how receptive they are to going through it.” – Interview 20.

In addition, six healthcare providers mentioned that they had limited capacity to address PA and fruit/vegetable consumption with their clients, and some linked it directly to COVID-19.

“So, I think from a primary care perspective, the biggest challenge that we’ve run into, again, unfortunately this year is going be COVID specific. At one point we only were open the one day a week, for example, so for me it’s a bit of a juggling act as far as coordinating the timing to make sure that my clients are still supported in their product as well as their follow up.” – Interview 15.

### Supports: supports needed or utilized by the healthcare providers to implement the intervention

Almost all the healthcare providers (n = 23) reported that they had supports in place to implement the PACE intervention. Specifically, allied health professionals/programs helped them implement the PA and fruit/vegetable consumption intervention. These healthcare providers also mentioned that the PACE intervention had provided an opportunity to refer their patients to supports that were already available at their clinics.

“I think … (the PACE intervention encourages) me to link them to the dietitian and our social worker and things like that.” - Interview 1.

“We have a physiotherapist here, we have dietitians, we have respiratory therapists, so… if someone tells me that they’re living on pancakes and smoothies for sustenance, I will suggest that maybe that’s not the best thing. If you’re smoking and having eight cups of coffee day, we try to deal with that, but anything beyond my [skills], I will refer to our registered dietitians.” – Interview 23.

However, even with these supports in place, 11 healthcare providers reported that there was a need for training about the PACE intervention, and six mentioned that there was a need for more resources with respect to the PA and fruit/vegetable consumption.

“I honestly would love to focus more on that, but it’s not enough information. I’m not trained enough, in a short period of time, I think, to give them information.” – Interview 2.

“I’m wondering if, when I say to somebody, are you interested in pursuing further, the relationship with diet and smoking, or with physical activity and smoking, if there was something besides just a tracking sheet to send them. Like basic dietary recommendations, or just a recommended level of physical activity, just to go along with that sheet. That might be something that would be helpful.” - Interview 14.

Several healthcare providers (n = 7) mentioned the lack of external supports or resources to increase PA or fruit/vegetable consumption during the COVID-19 pandemic, for example, the closure of gyms, exercise programs or cooking classes.

“So that is one service our health centre offers is like, different kind of walking groups that they do, and different exercise groups. And then our dietitian does do some cooking demonstrations in groups previously to COVID, but since COVID there’s been less of that.” – Interview 22.

### Usability: the intervention being clearly defined, core features being well operationalized, and any adaptations needed for the context of the population

Most healthcare providers felt that the PACE intervention was well-defined but some adaptations were needed. Fifteen healthcare providers mentioned using their own approach when implementing the PACE intervention, for example, using the Health Promotion 6 Pack or asking clients to use journals rather than tracking sheets.

“So, what I recall is that it says… do you want to provide your own intervention, right? I also will do my own just because of the nature of Wraparound care here at the CHC. And so, often if there’s an issue about food, or if there’s an issue about exercise, I like to try to keep it with providers that are able to make more contact with the patient on a regular basis. So, often, I will do my own.” - Interview 24.

A majority of healthcare providers (n = 19) reported that the resources were not used by most of the clients, even though clients appreciated receiving them. Very few clients used or returned the tracking sheets for PA and fruit/vegetable consumption.

“So, I haven’t had anybody actually want that. I’ll go over it, I’ll say you know, the STOP Program has asked a question here, do you want this self-monitoring, self-tracking? And they’re pretty aware already. They say, ‘I know I have low levels of fruits and vegetables, I know I should eat more, but I just don’t.’ And like, same with the exercise. ‘I know I should exercise, but I don’t do as much as I should.’ So, it always ends up being for me like, client made aware but declined.” - Interview 25.

“I think it’s well appreciated. Again, I’m not seeing them come back… They receive it well and I would say about 50% are coming back and saying ‘this is how it’s been happening. I have been monitoring this, I have been trying to do this.’ So, it’s useful to the extent that it can be with this population.” – Interview 23.

Several healthcare providers (n = 14) mentioned the need for a tracking sheet that could be easily provided to the patients, for example, without the barrier of printing them.

“I would like some things physically printed out already, because when I was printing things…black and white is not engaging. It’s not interesting, because I don’t have a colour printer.” – Interview 17.

A few healthcare providers (n = 8) commented on the prompts and mentioned that PACE intervention was clearly defined and easy to use.

“With the PACE Intervention itself, it’s a very nice prompting system. I like how it’s organized, as far as when you’re putting in that information, it’s giving you that follow up immediately. It doesn’t re-direct you to something like another window, for example, or a separate way to look it up. It simply says, you know, this is what we have noticed based on the answers you’ve given for the specific client. And then I really like that it does provide you that resource directly, it’s a very tangible piece that I could print off for a client and give them (during) enrollment.” – Interview 15.

“I think because they are so quick…I think that they’re quite accessible I find. I feel like they’re nicely presented, they’re colourful. You know, it’s an easy read… I think they’re a good quick reference that reminds you that any opportunity can kind of be a teachable opportunity, or teachable moment I guess, especially to provide patient education about different things, and it doesn’t have to be an hour-long spiel for me about the impacts of diet and exercise on whatever sort of chronic disease we’re talking about. So, I think probably the ease and ability to read and access that, like, their accessibility, to me, as a provider is key.” – Interview 18.

Some healthcare providers (n = 8) reported that the COVID-19 pandemic created barriers for the usability of the intervention, since the program had to move from in-person service to virtual/phone based services. The virtual appointments made it more difficult to engage patients/establish rapport or provide resources to them.

“Um, well now that we’re doing it over the phone, I mean, it’s not as personable… I don’t find you get the same rapport with your patient, and maybe they will tell you more in person than they would over the phone as far as other things.” – Interview 2.

On the other hand, some healthcare providers (n = 5) mentioned pandemic-related facilitators of the implementation. For example, phone appointments allowed some patients to be more comfortable communicating about low PA or fruit/vegetable consumption.

“It’s made me realize that this is easily done over the phone and people were more willing to do it I think, because… when it’s face-to-face I think they’re a little bit more ashamed of themselves and they open up, I find, a little bit more when they’re on the phone.” – Interview 1.

Several healthcare providers reported that offering the intervention on a case-by-case basis (n = 20) or adapting the intervention based on the clients’ needs (n = 17) facilitated the implementation of the intervention. For example, healthcare providers would offer the intervention if they perceived their clients were more receptive to changing their PA or fruit/vegetable consumption. Some of these healthcare providers also mentioned a need for change in the language or measurement of PA or fruit/vegetable intake or modifying the question based on clients needs.

“So, sometimes it’s not about necessarily meeting all of those recommendations we provide them with, but rather meeting them where they’re at to see what their food security looks like, what they could potentially have access to, and doing the best that we can to support them in accessing additional resources… And sometimes it’s that modification of if you are buying food at the Dollar Store, is there some healthy options there? Is there a way that we can kind of look at labels even, to see if, you know, salt intake can be lessened, or just more simple attainable things rather than, can you go and physically afford those vegetables and fruits that unfortunately might be unattainable at this time?” - Interview 15.

“So mobility and frailty is all stuff that will influence what message I will more put emphasis on and not… Some patients are not able to ambulate as freely as they should or they could, so sometimes I kind of put the emphasis on physical strength, or strength training, so that’s where, you know, I kind of go with the flow, or play around those types of areas.” – Interview 19.

“I think the vigorous, strenuous activity is, um…I don’t like the wording. I think if they were just, like, even a light…what kind of physical activity level would you rate yourself at, like as a zero, to three, a three to five, a five to eight, or a nine to 10, type of level, um, person, you know. And I don’t know if that would mean anything because it’s kind of vague but at the same time, asking people for how many days a week that the exercise and how many minutes, doesn’t seem…like, yes, they want to aim for a certain amount, I get that, but at the same time, I don’t know if that’s too intimidating for some people” – Interview 2.

In addition, 15 healthcare providers reported that the PACE intervention may be more easily implemented at a follow up visit, since the intake appointment may have too much information provision.

“I feel like it adds a bit too much to the intake. You’re already talking a lot and asking a lot and we have limited time with our patients, so I feel like sometimes I kind of skip over it a bit too quickly because you’re trying to rush it into an already really long intake appointment.” – Interview 12.

“The follow up visits are really quick, so I think if we had, perhaps maybe more time and maybe that’s we can explore more of these fruits and vegetables and exercise, and that might prompt more conversation.” – Interview 2.

### Triangulation of quantitative and qualitative findings

A majority of healthcare providers (n = 17) reported that randomization did not impact their implementation of the PACE intervention, i.e., healthcare providers would address low PA and fruit/vegetable consumption irrespective of receiving the prompt at the end of the intake questionnaire.

“I think I’m just so used to, again, with my background with health promotion, when we’re going through those questions, it just kind of registers in my mind if their physical activity is low, or their fruits and vegetable intake is low, so I would treat every patient the same.” – Interview 10.

In addition, 12 healthcare providers mentioned that the PACE intervention improved client satisfaction, however, 19 of healthcare providers mentioned that the intervention did not have an effect on smoking cessation.

“I think for the people that it makes sense for and are receptive, they probably appreciate the overall kind of holistic approach that it’s taking versus, like, you’re going to come in and I’m going to put you on the patch and then we’re going to decrease your patch and then you’re done.” – Interview 12.

“I wouldn’t say that’s a huge factor in success or failure (of smoking cessation). I wouldn’t say it’s changed anything at all.” – Interview 16.

## Discussion

In this qualitative study, we utilized the Hexagon Tool to investigate factors influencing the implementation of an intervention aimed at promoting PA and fruit/vegetable consumption among patients enrolled in a smoking cessation program delivered in primary care. The data from the six domains of the Hexagon Tool can be organized into three broader areas of healthcare provider related factors, patient related factors, and COVID-19 pandemic related factors as described below.

Most healthcare providers reported that addressing PA and fruit/vegetable consumption was needed by, and a good fit for, most of the STOP program patients. Healthcare providers mentioned examples of exercise helping in reducing cravings or reducing post-cessation weight gain, and the need for a holistic approach to help a patient quit smoking. Previous literature has shown similar findings about the relevance of exercise and diet to smoking cessation [16, 46–48]. However, according to healthcare providers, patients may not always prioritize exercise and diet while their focus is on quitting smoking. Similarly, a study by Dolor et al. (2010) [34] found that healthcare providers reported patients might not be interested in hearing about diet and exercise; however, patients were more comfortable discussing these modifiable health risk behaviours. This finding provides some understanding as to why, in the RCT we conducted, we found no effect of the intervention on patients’ smoking status, PA, or fruit and vegetable consumption at 6 months [26]. In addition, this finding also indicates a need for future research to explore patient’s perspectives during the delivery of the PACE intervention.

Several patient-related factors impacted the implementation of the intervention, including social determinants of health such as income, education, food insecurity; mental/physical health; and patients’ ability or motivation to use the tracking sheets and to increase their PA and fruit/vegetable consumption. However, asking patients about these behaviours helped healthcare providers open up the conversation with patients during the intake appointment; and the conversation could be continued during the follow up appointments based on these patient related factors. In addition, external resources in the form of referrals to allied health programs/professionals or practical information about how to increase exercise and fruit/vegetable consumption facilitated the implementation of the intervention. Specifically, considering the social determinants of health, healthcare providers needed more information about resources for those patients who couldn’t afford to purchase fruit/vegetable or go to the gym for exercise. In addition, the population of clients who accessed support at the STOP program have been known to belong to lower income groups, highlighting the importance of such considerations in these client populations [49]. These considerations have been explored by others [50]. Furthermore, there is a need to explore strategies to address such barriers through future research studies. This finding, showing among those providers who implemented the intervention, how they decided who to deliver the intervention to, helps us contextualize some of the quantitative results. In the RCT, we saw that healthcare providers delivered the intervention to approximately 60% (1083/1675) of their patients [26].

The COVID-19 pandemic and associated public health measures affected the healthcare system [51, 52] and the population [53, 54] in general, and this study found how it also affected the implementation of the PACE intervention. Healthcare providers mentioned several pandemic related factors affecting the implementation, such as closure of exercise/diet facilities/programs, transition to virtual appointments and associated change in rapport, social and mental health impacts of the pandemic, etc. Previous research has similarly shown the impact of the pandemic on diet and exercise [55], mental health [56], and format of healthcare delivery [52]. This study helped understand the impact of this unintentional contextual factor on the delivery of an intervention to increase PA and fruit/vegetable consumption. Future research studies need to qualitatively explore the impact of such unintentional factors affecting the implementation of programs.

### Limitations

The recruitment of participants was limited to a few STOP healthcare providers who agreed to participate in the study, and may not reflect the views of those who did not participate. However, an effort was made to recruit healthcare providers from diverse settings (e.g., FHTs, CHCs) to obtain varied perspectives. Similarly, the framework analysis approach allowed the research team to conduct a content analysis of the qualitative data, but these results are not generalizable. The Hexagon Tool may not be able to directly capture other implementation factors such as self-efficacy among healthcare providers. However, the benefit of utilizing the framework analysis approach is the ability to use a pre-existing theoretical framework to conduct deductive analysis, while allowing new codes to be recognized using inductive analysis, leading to a comprehensive qualitative analysis of the data. This study elicited the perspectives of healthcare providers, and there may be a need to elicit patients’ feedback about the intervention. However, the findings from this research study can be used to address the barriers to implementation at the healthcare provider level.

## Conclusion

This study adds to the existing literature on multiple health risk behaviour interventions. It helps to understand the complexities of implementing an exercise and diet intervention within a smoking cessation program delivered in primary care setting. It may be beneficial to adopt a tailored approach to address exercise and diet, depending on the patient’s life circumstances. This may include an emphasis on why such a holistic approach is important, and then identifying and attempting to resolve patients’ social determinants of health and mental/physical health which may contribute to low levels of PA and fruit/vegetable consumption. In addition, training, external resources and support from allied health programs are needed to complement healthcare providers in these efforts.

### Electronic supplementary material

Below is the link to the electronic supplementary material.

Supplementary Material 1

Supplementary Material 2

## Data Availability

The qualitative data from the current study is not publicly available to protect the privacy and confidentiality of the healthcare providers, however, representative quotes from the themes have been included to provide context of the analyzed data.

## Abbreviations

PA: Physical Activity
PACE: Promoting and Accelerating Change through Empowerment
STOP: Smoking Treatment for Ontario Patients
RCT: Randomized Controlled Trial
FHT: Family Health Team
CHC: Community Health Centre
NPLC: Nurse Practitioner-Led Clinic
