# Supplementary material for: Healthcare providers’ perspectives on implementing a brief physical activity and diet intervention within a primary care smoking cessation program: a qualitative study
Source: BMC Prim Care. 2024 Jan 6;25:16. doi: 10.1186/s12875-023-02259-3 (PMC10770944; doi:10.1186/s12875-023-02259-3)
Supplement: Supplementary file 1 — Supplementary Material 1 [file 12875_2023_2259_MOESM1_ESM.docx]

**Appendix J: Follow-up Interview Guide (Evaluation)**

*The purpose of the interview is to learn about the experience of your clinic in delivering the PACE intervention as part of the* ***STOP program,*** *facilitators and challenges to program delivery, including the onset of the COVID-19 pandemic, and outcomes of the program. The PACE Intervention refers to the prompts that appear on the STOP Portal for addressing diet and physical activity with patients.*

**If you have not already consented to participate in this study**, have you had a chance to review the informed consent form that was emailed to you prior to this interview?

IF YES Do you have any questions for me regarding this interview?
Do you give your consent to participate in this interview?
Do you give your consent to be audio recorded?

IF NOT Ok, I’m now going to review the informed consent document with you

And if you haven’t already done so, please return the consent form by scanning and emailing it to sheleza.ahad@camh.ca

*Ice Breaker*

1. Can you tell me about your role at your agency and how long you have been involved in STOP?

*Research Questions*

1. What training, if any, have you received for addressing multiple modifiable risk factors?
   1. Probe for any training in addressing physical activity and fruit/vegetable intake
2. Can you tell me about your experiences using the STOP portal since the adaptation to provide a brief intervention and self-monitoring resources (paper-based daily trackers) to clients was launched?
   1. Gauge for positive vs. negative overall impression, ease of identifying patients with other modifiable risk factors (e.g. alcohol use, mood, physical activity, diet), how useful (or not) the self-monitoring resource is.
   2. Probe for how their experience using the STOP portal may have changed after the onset of the COVID-19 pandemic (March 2020)
   3. Probe for practitioner’s perspectives on various iterations of the CDSS pathway in STOP (alcohol only, alcohol and mood, all 4 risk factors).
      1. If practitioner doesn’t remember or joined STOP recently, probe for their perspectives on addressing these risk factors
3. How has this adaptation impacted your current workflow in the STOP program? In your practice in general? Please describe any changes before and during the COVID-19 pandemic
   1. Probe: Has adaptation affected length of time to complete baseline? Has adaptation impacted how many new patients are seen? Has adaptation impacted how many patients return for follow-up visits?
   2. Did HCP notice that some patients were prompted to receive the PACE intervention, while others (who presented multiple modifiable risk factors) were not? Did you provide an intervention even when you were not prompted? If so, how do you decide on when to provide this intervention?
4. What factors influenced your decision to deliver the PACE intervention as part of the ***STOP program*** at your clinic?
5. Probe (ask about any changes following the onset of COVID-19):
   - 1. Perceived needs of clients ; can you describe any changes to client needs after the COVID-19 Pandemic? (i.e., changes in diet and physical activity)
     2. Motivation and perceived clinical responsibility to deliver the intervention as part of smoking cessation treatment; Describe how COVID-19 increased/decreased your perceived obligation/motivation to address modifiable risk factors with clients?
     3. Fit (adaptation fits with other initiatives happening in the clinic)
     4. Evidence for addressing modifiable risk factors (there is strong evidence to implement the change)
     5. Resource availability (portal functions adequately, resources are easily available, staff is trained, staff is available to perform tasks); Describe any changes in resource availability that influenced the delivery of the intervention following the COVID-19 pandemic
     6. Capacity to implement (sufficient staff to deliver the program; staff knowledge/training/skills in addressing multiple modifiable risk factors, equipped to improve implementation over time); describe your capacity to implement before and following the COVID-19 pandemic
        - Do you feel you have the skills required to deliver the PACE intervention?
6. What challenges and barriers to delivering the PACE intervention as part of the ***STOP program*** did you encounter? How did you address these challenges or barriers? Please explain.
   1. Probe: *prior to the COVID-19 pandemic, can you describe any challenges/barriers related to the following*
      1. Organizational: lack of time, resources, support, funding; other priorities; technical difficulties
      2. Practitioner-level: lack of staff confidence, knowledge, training
      3. Patient-level: complex patient cases, mental health issues, other addictions, patients not ready to quit smoking and change other health behaviours, patient not interested in receiving the self-monitoring resources.
      4. Program specific challenges: question was annoying, resources were hard to find, etc.
   2. Probe: changes in any of these challenges/barriers following the onset of COVID-19
7. How might we be able to improve this adaptation for you?
8. How might we be able to improve this adaptation for clients?
9. How are your clients responding to the brief interventions? Self-monitoring resources?
10. Probe for clients who have a different numbers of risk factors, different socioeconomic statuses, genders.
11. Probe for client response to physical activity and fruit/veg intake.
12. How do you feel about the self-monitoring resources (provided through the STOP Portal, for encouraging clients to make changes to their modifiable risk factors and/or quitting smoking)?
    1. Probe for clients who drink at different levels, different levels of physical activity, levels of fruit/veg intake, with comorbidities, income, sex/gender
    2. How could they be improved?
13. Thus far, what changes have you observed in smoking cessation rates at your clinic as a result of delivering the PACE intervention as part of the ***STOP program***?
    1. Probe for changes before/during COVID-19
14. Thus far, what changes have you observed, if any, in client satisfaction at your clinic as a result of delivering the PACE intervention as part of the ***STOP program***?
    1. Probe for client’s satisfaction around to discussing/addressing physical activity and fruit/veg intake.
    2. Probe for changes in satisfaction before/during COVID-19
15. Did this initiative feel relevant for your clients in the STOP program?

*Closing Question*

1. Is there any other advice that you would like to provide for us regarding this initiative and the STOP portal adaptation?
