# Supplementary material for: Healthcare providers’ perspectives on implementing a brief physical activity and diet intervention within a primary care smoking cessation program: a qualitative study
Source: BMC Prim Care. 2024 Jan 6;25:16. doi: 10.1186/s12875-023-02259-3 (PMC10770944; doi:10.1186/s12875-023-02259-3)
Supplement: Supplementary file 2 — Supplementary Material 2 [file 12875_2023_2259_MOESM2_ESM.docx]

**RATS guidelines for qualitative manuscript**

| **Criteria** | **Location in the manuscript** |
| --- | --- |
| R - Relevance of study question:   - Is it important for medicine or public health? - Is the research question explicitly stated? - Is it linked to existing knowledge base (literature, theory, practice)? - Is the research question justified? | Page 2  Page 3  Page 2-3  Page 2-3 |
| A - Appropriateness of qualitative method:   - Is qualitative methodology the best approach for the study aims? - Is the study design justified? - Why was a particular method (for example, interviews) chosen? | Page 3  Page 3  Page 3 |
| T - Transparency of procedures:  Sampling:   - Are criteria for selecting the study explained and justified? - Why were these participants selected as the most appropriate to provide access to type of knowledge sought by study?   Recruitment:   - How and by whom was recruitment conducted? - Was selection bias discussed? - Who chose not to participate and why?   Data collection   - Was collection of data systematic and comprehensive? - Are methods explicitly outlined and examples, such as interview questions, given? - Are characteristics of study group and setting clearly described? - When was data collection stopped and why?   Role of researchers:   - Do the researcher(s) critically examine their own influence on the formulation of the research question, data collection, and interpretation? - Do the researchers occupy dual roles (clinician and researcher)?   Ethics:   - Is informed consent detailed? - Is a discussion of anonymity and confidentiality presented? - How were anonymity and confidentiality ensured? - Was approval from ethics committee received? | Page 7  Page 7  Page 3  Page 9  Page 3-4  Page 3  Page 3-4, Additional File 1  Table 1  Page 3  Page 4  Page 4  Pages 3 and 10  Pages 3 and 10  Pages 3 and 10  Pages 3 and 10 |
| S - Soundness of interpretative approach:   - Is process of analysis described in-depth? - How were themes derived from the data? Were alternative explanations sought? - Were negative or deviant cases analysed? - Are the interpretations clearly presented and adequately supported by the evidence? - Were quotes used and on what basis were these chosen? - Was trustworthiness of data checked? - Was an audit trail or triangulation employed? - Are findings presented with reference to existing theoretical and applied literature? - Are limitations discussed? | Page 3-4  Page 3-4  Page 3-4  Page 4-9  Pages 4 and 9-20  Page 4  Page 3-4  Page 9  Page 9-10 |
